# Laying hen responses to balanced protein reduction on performance, egg quality, nitrogen balance, and fat and mineral utilization

**DOI:** 10.1093/jas/skaf465

**Published:** 2026-01-06

**Authors:** Elijah Ogola Oketch, Myunghwan Yu, Shan Randima Nawarathne, Nuwan Chamara Chathuranga, Jeseok Lee, Haeeun Park, Bo Keun Lee, Kwan Eung Kim, Jung Min Heo

**Affiliations:** Department of Animal Science and Biotechnology, Chungnam National University, Daejeon, 34134, Republic of Korea; Department of Animal Science and Biotechnology, Chungnam National University, Daejeon, 34134, Republic of Korea; Poultry Research Center, Department of Animal Resources Development, National Institute of Animal Science, Rural Development Administration, Pyeongchang, 25342, Republic of Korea; Department of Animal Science and Biotechnology, Chungnam National University, Daejeon, 34134, Republic of Korea; Department of Animal Science and Biotechnology, Chungnam National University, Daejeon, 34134, Republic of Korea; Department of Animal Science and Biotechnology, Chungnam National University, Daejeon, 34134, Republic of Korea; Department of Animal Science and Biotechnology, Chungnam National University, Daejeon, 34134, Republic of Korea; Nonghyup Feed Inc, Seoul, 05398, Republic of Korea; Nonghyup Feed Inc, Seoul, 05398, Republic of Korea; Department of Animal Science and Biotechnology, Chungnam National University, Daejeon, 34134, Republic of Korea

**Keywords:** abdominal fat, egg quality, laying performance, nitrogen excretion, protein, tibia

## Abstract

The effect of graded reductions in balanced crude protein (**CP**) on hen productive performance, egg quality, nitrogen balance, abdominal fat deposition, tibia traits, and relative economic outcomes from 26 to 44 wk of age (**WOA**) was investigated. A total of 252 26-wk-old Hy-Line Brown hens were housed in enriched cages (seven birds/cage) and randomly allocated to one of four different dietary CP levels of iso-energetic diets with nine replicates per treatment. The trial was conducted over two phases of 26 to 34 and 36 to 44 WOA. Diets included a high-protein (**HP**; 18.0% and 17.0% CP in Phases 1 and 2), medium-protein (**MP**), low-protein (**LP**), and very low-protein (**VLP**) series, representing stepwise reductions of 0.50, 1.00, and 1.50 percentage points relative to HP. Limiting amino acids (**AA**; lysine, methionine, and threonine) were supplemented to ensure balanced AA profiles. Collected data were analyzed using the general linear model (GLM) procedure for one-way ANOVA; statistical significance was set at *P* < 0.05, and trends were noted at 0.05 < *P* < 0.10. Balanced protein reduction tended to improve abdominal fat contents (2.66% to 2.85%; *P* = 0.059), but reduce body weight gain (141.66 to 95.66 g; *P* = 0.089), particularly with the VLP diet. Across 26 to 44 WOA, graded CP reduction lowered egg weight (60.36 to 59.40 g; *P* < 0.05) and feed conversion efficiency (1.93 to 1.97 g feed/g egg; *P* < 0.05); and tended to reduce egg mass (57.02 to 55.11 g/hen/day; *P* = 0.080), particularly in the VLP group. As to egg quality, Haugh units were higher (*P* < 0.05) with HP and MP diets (94.60 and 94.30) than LP and VLP diets (93.66 and 93.04) across 26–44 WOA. In contrast, LP and VLP diets tended to improve yolk color (8.38 to 8.49; *P* = 0.076) and egg-breaking strength (5.39 to 5.51 kg; *P* = 0.058) across 26–44 WOA. Dietary CP reduction linearly reduced nitrogen consumed and excreted by more than 10% (*P* < 0.05). Tibia-breaking strength tended to decline with dietary CP reduction (*P* = 0.094), decreasing from 27.62 kg in HP to 25.54–25.68 kg in the LP and VLP diets. Economically, reduced CP lowered egg income (*P* < 0.05) at weeks 34 and 44 (2.00 to 1.77$; 1.96 to 1.89$, respectively); and feed costs at week 34 only (0.54 to 0.52$; *P* = 0.088), but profit margins remained unaffected (*P* > 0.10). Conclusively, these results confirm the effectiveness of balanced dietary protein reduction in maintaining egg production rate and most egg quality traits while reducing nitrogen excreted and feed costs.

## Teaser

Reduced-protein diets supplemented with essential amino acids were effective in reducing feed costs and nitrogen excreted without adversely affecting hen performance and egg quality.

## Introduction

Protein is essential for growth, development, health, reproduction, and productivity (Baker, 2009; Alabi and Adedokun, 2025). Birds require precise levels and balance of both dispensable and non-dispensable AA. However, 18%–20% of dietary protein may escape complete digestion (Ndazigaruye et al., 2019), leaving undigested protein in the hindgut for undesirable fermentation that could compromise hen health (Elling-Staats et al., 2022). Increased dietary protein intake is also associated with greater nitrogen and water excretion, raising litter moisture and environmental stress (Swiatkiewicz et al., 2017; Song et al., 2025). Elevated litter moisture, due to higher water intake relative to feed intake, is associated with higher footpad dermatitis risk, ultimately compromising paw quality and bird welfare (Woyengo et al., 2023; Son et al., 2024). Additionally, higher protein diets could favor the proliferation of pathogenic microbes over beneficial ones, causing gut dysbiosis (Apajalahti and Vienola, 2016; De Cesare et al., 2019; Cardoso Dal Pont et al., 2020).

Reducing dietary protein is collectively targeted to improve animal health and welfare while reducing feed costs and nutrient excretion into the environment, without compromising the need to maximize the genetic potential of the modern laying hen (de Persio et al., 2015; Wang et al., 2017). Importantly, protein supply constitutes the second most expensive feed component after energy. Historically, laying hen diets were often formulated with higher CP and AA contents in the hopes of maximizing egg size and number, but advances in nutrition have made it possible to formulate more precisely (Applegate and Angel, 2008; Toghyani et al., 2024). Precise formulation using digestible AA estimates and ideal ratios aims to optimize protein deposition while reducing nitrogen excretion. As a result, research has shown the capacity of low-protein diets with supplemental crystalline AA to reduce nitrogen excretion without affecting laying performance and egg quality (Ji et al., 2014; Heo et al., 2023). Contrarily, further reducing dietary protein beyond a certain point reduced laying hen performance and egg quality (Shim et al., 2013; Rozenboim et al., 2016; Liu et al., 2024).

It is also appreciated that nutrients exist in a complex matrix involving starch and non-starch polysaccharides, proteins, lipids, minerals, and vitamins; thus, nutrient metabolism is interconnected (Oketch and Heo, 2025a). Consequently, a modulation of dietary protein levels can affect the metabolism of other feed components, including lipids and minerals. In this regard, reducing dietary protein disrupts protein to energy balance, increasing energy concentration and consumption relative to protein (Woyengo et al., 2023). This energy/protein imbalance is often associated with higher abdominal fat deposition, potentially compromising productivity and liver health, more so in older hens (Hu et al., 2024; Chathuranga and Heo, 2025). Even though mineral utilization is integral to hen performance and egg quality (Elnesr et al., 2024; Waters et al., 2024), the modulatory effects of balanced dietary protein levels on mineral metabolism remain insufficiently studied. Recent findings by Zhang et al. (2025) highlight this gap, showing that dietary CP significantly affected both laying performance and mineral utilization. While reducing dietary CP to 15.0%–15.5% enhanced protein digestion and Mn utilization and deposition, but compromised the laying performance; increasing dietary protein to 17.5% enhanced the deposition of Ca and P in the tibia (Zhang et al., 2025).

To satisfy genetic potential and maximize productive capacity, dietary CP and AA contents and their balance in the feed are continuously updated by breeder companies (Hy-Line Brown Commercial Layers, 2018, 2024), presenting an opportunity for their constant evaluation in hen feeding regimes. It is the authors’ observation that scant information is available exhibiting whether these revised specifications can be reduced in efforts to maintain performance and egg quality while reducing nitrogen excretion into the environment and feed costs. The current study was therefore conducted to investigate the influence of graded levels of balanced dietary protein reduction on laying performance, egg quality, nutrient excretion, abdominal fat contents, economic performance, and some tibia traits of Hy-Line Brown laying hens from 26 to 44 WOA. It was hypothesized that balanced dietary CP reduction could maintain the laying performance and egg quality while reducing nitrogen excretion and feed costs of laying hens from 26 to 44 WOA. Reflecting potential effects on mineral and fat metabolism, modulatory effects of dietary protein levels on some tibia traits and relative abdominal fat contents, respectively, were also examined.

## Materials and Methods

### Animal ethics statement

The experiment was carried out at the Poultry Unit, Cheongyang Research Facility of Chungnam National University, following the approved experimental protocol and procedures stipulated by the Animal Ethics Committee (Protocol Number: 202407A-CNU-125).

### Birds and housing

A total of 252 Hy-Line Brown hens (24-wk-old) were purchased from a commercial supplier (Ganongbio, Pocheon-si, Republic of Korea). The birds were individually weighed upon arrival to verify their body weight, and then randomly allocated to 36 enriched cages (7 birds per cage). Hens underwent a 2-wk adaptation period and were equally fed one of the four dietary treatments, with the laying rate being monitored. Cages (90 × 90 × 70 cm) included enrichments per standards (European Commission, 1999). A lighting regimen of 16 h of continuous light and 8 hours of darkness was maintained in a windowless and temperature-controlled facility (20–26 °C; 45%–50% humidity). Each cage was equipped with four nipple drinkers and a removable front feeder. Bird health, welfare, mortalities, water supply, temperature, lighting, humidity, and ventilation were routinely monitored.

### Dietary treatments

Experimental diets were formulated to be iso-energetic and adjusted over two phases: Phase 1 (26–34 wk) and Phase 2 (36–44 wk) based on feed intake, egg production, and hen age. Four experimental diets were formulated to have a 0.50 percentage points reduction of dietary protein (Table 1). Four treatment series were established in each phase: high protein (HP; 18.00% and 17.00%), medium protein (MP; 17.50% and 16.50%), low protein (LP; 17.00% and 16.00%), and very low protein (VLP; 16.50% and 15.50%), for Phases 1 and 2, respectively. Limiting AA, including lysine, methionine, and threonine, were supplemented to ensure balanced AA profiles in line with breeder recommendations (Hy-Line Brown Commercial Layers, 2024). Feed samples were collected and evaluated to ascertain proximity to formulated targets for gross energy (bomb calorimeter), crude protein (990.03; AOAC, 2007), crude fat (920.39; AOAC, 2007), and ash (942.05; AOAC, 2007).

**Table 1.** Ingredients and the calculated and analyzed nutrient composition of the dietary treatments

Ingredients	26–34 WOA				36–44 WOA			
	HP	MP	LP	VLP	HP	MP	LP	VLP
	**18.0%** **CP**	**17.5%** **CP**	**17.0%** **CP**	**16.5%** **CP**	**17.0 %** **CP**	**16.5%** **CP**	**16.0%** **CP**	**15.5%** **CP**
**Corn**	57.97	59.04	60.16	61.30	59.60	60.74	61.82	62.95
**Wheat bran**	3.78	3.22	2.64	2.04	4.20	3.62	3.04	2.44
**Soybean meal, 44%**	19.32	20.14	20.94	21.76	18.18	18.98	19.80	20.62
**Corn gluten**	5.90	4.56	3.22	1.84	5.00	3.64	2.30	0.94
**Soybean oil**	1.00	1.00	1.00	1.00	0.80	0.80	0.80	0.80
**Dehydrated salt**	0.31	0.31	0.31	0.31	0.31	0.31	0.31	0.31
**Mono-calcium phosphate**	1.27	1.27	1.27	1.27	1.13	1.13	1.14	1.14
**Limestone**	9.82	9.82	9.81	9.80	10.15	10.14	10.13	10.13
**DL-Methionine**	0.14	0.17	0.19	0.21	0.14	0.16	0.19	0.21
**L-Lysine**	0.12	0.11	0.10	0.09	0.12	0.11	0.10	0.09
**Threonine**	0.00	0.00	0.00	0.01	0.00	0.00	0.01	0.02
**Choline chloride**	0.07	0.07	0.06	0.06	0.08	0.07	0.07	0.06
**Vitamin-mineral premix**^1^	0.30	0.30	0.30	0.30	0.30	0.30	0.30	0.30
**Calculated nutrient composition, %**								
**ME, kcal/kg**	2700	2700	2700	2700	2680	2680	2680	2680
**Crude protein**	18.00	17.51	17.00	16.49	17.01	16.49	16.00	15.50
**Crude ash**	13.39	13.38	13.37	13.36	3.14	3.13	3.12	3.11
**Crude fiber**	2.50	2.49	2.48	2.47	2.44	2.43	2.42	2.41
**Crude fat**	3.52	3.52	3.51	3.51	13.53	13.52	13.51	13.50
**Calcium**	4.00	4.00	4.00	4.00	4.10	4.10	4.10	4.10
**Total P**	0.59	0.59	0.59	0.59	0.56	0.56	0.56	0.56
**Available P**	0.35	0.35	0.35	0.35	0.32	0.32	0.32	0.32
**Linoleic acid**	1.68	1.68	1.68	1.68	1.50	1.49	1.49	1.49
**Calculated SID amino acid values**								
**Lysine**	0.78	0.78	0.78	0.78	0.74	0.74	0.74	0.74
**Methionine**	0.43	0.44	0.45	0.46	0.41	0.42	0.43	0.43
**Methionine + cysteine**	0.70	0.70	0.70	0.70	0.67	0.67	0.67	0.67
**Threonine**	0.57	0.56	0.55	0.55	0.54	0.53	0.52	0.52
**Tryptophan**	0.16	0.16	0.16	0.16	0.15	0.15	0.15	0.15
**Valine**	0.70	0.69	0.69	0.68	0.67	0.66	0.66	0.65
**Analyzed values**								
**Crude protein**	18.27	17.84	17.34	16.89	17.20	16.73	16.27	15.79
**Crude fat**	3.76	2.14	3.24	3.04	4.54	4.74	4.73	4.86
**Crude fiber**	2.29	2.26	2.30	2.37	2.00	1.64	1.99	2.00
**Crude ash**	13.61	12.50	12.17	12.91	11.60	13.17	12.79	11.06
**Gross energy**	3612	3801	3738	3754	3499	3591	3811	3716

1 Provided per kilogram of diet: vitamin A (trans-retinyl acetate), 3,900,000 IU; vitamin D_3_ (cholecalciferol), 1,500,000 IU; vitamin E (DL-α-Tocopherol acetate), 6200 IU; vitamin B1 (thiamin), 4.4 mg; vitamin K3 (menadione), 330 mg; vitamin B2 (riboflavin), 2600 mg; vitamin B5 (D-pantothenic acid), 2500 mg; vitamin B3 (nicotinic acid), 11,200 mg; vitamin B4 (choline), 140,000 mg; and vitamin B9 (folic acid), 275 mg; vitamin B6 (pyridoxine), 550 mg; vitamin B1 (thiamine), 440 mg; vitamin B7 (biotin), 12 mg; Fe (from iron sulfate), 90 mg; Cu (from copper sulfate), 8.8 mg; Zn (from zinc oxide), 40 mg; Mn (from manganese oxide), 54 mg; I (from potassium iodide), 0.35 mg; Se (from sodium selenite), 0.30 mg.

### Growth and productive performance

All hens were separately weighed at weeks 26, 34, 36, and 44 to evaluate body weight. Productive performance was assessed every 4 wk during Phase 1 (26–34 wk) and Phase 2 (36–44 wk). The total egg number and weight were recorded daily. Hen feed intake was adjusted per Hy-Line (2024) guidelines and calculated weekly as the difference between feed offered and feed refused. Egg production was expressed as hen-day egg production (**HDEP**), while egg loss percentages considered damaged eggs as a fraction of the total eggs laid. Damaged eggs included those judged to be dirty, rough, misshapen, cracked, white-banded, pale-shelled, soft-shelled, or shell-less. Feed conversion ratios (**FCR**) and egg mass were evaluated using the HDEP, feed intake, and egg weight data (Oketch et al., 2025b).

### Internal egg and eggshell quality

At weeks 30, 34, 40, and 44, a total of 90 randomly selected and undamaged eggs (10 eggs/cage) were evaluated for egg quality. Internal quality (albumen height, Haugh units, yolk color, yolk, and albumen percentages) and shell quality (specific gravity, shell color, breaking strength, shell thickness, and shell percentage) were completed within 24 h. A texture analyzer (TA.XTplusC, Stable Micro Systems, Surrey, England) was used to gauge the shell-breaking strength. Internal quality and shell color were measured using an egg multitester instrument (TSS QCM+ Range, York, England) featuring an internal Haugh Unit calculator, digital balance, shell color reflectometer, albumen height gauge, and yolk colorimeter. Albumen height was read at least 1 cm from the yolk (Jones, 2012), and yolk color was measured against the DSM yolk color fan (1, light yellow; 15, orange). Specific gravity was determined by immersing eggs in a series of saline solutions with increasing densities (Oketch et al., 2025). Yolk, albumen, and shell were separated and weighed to calculate their percentages relative to egg weight. Absorbent paper was used to remove adhering albumen before eggshells were dried for 24 h to determine their percentage. Shell thickness (including membranes) was measured at the sharp, equator, and blunt ends using a micrometer (Digimatic MDC-MX Series, Illinois 60502, USA).

### Abdominal fat contents and some tibia characteristics

At the end of the experiment, one hen per cage (nine per treatment; 36 in total) was sacrificed by carbon dioxide asphyxiation for abdominal fat contents and tibia analyses. Tibia samples from the left and right legs were defleshed, dried, and analyzed for weight, length, volume, and density following Oketch et al. (2024). Abdominal fat was collected, weighed, and expressed as a percentage of body weight. Tibia breaking strength (tensile or compressive load required for fracture, kgf) was then measured using a texture analyzer (Lloyd TA1 Series Texture Analysis Machine, AMETEK Measurement and Calibration Technologies, Florida 33773, USA).

### Nitrogen balance

Excreta samples were collected at weeks 30 and 40 using all nine replicates (seven birds/replicate cage). Pooled excreta samples were stored at −20 °C and oven-dried at 45 °C for 96 h before fine-grinding for analyses. The egg nitrogen content is estimated to be 1.936; 1.936 is derived from the determined protein content of an egg at around 12.10 (Miranda et al., 2015), which was then divided by 6.25. Subsequently, the nitrogen intake, excretion, total nitrogen retained (**TNR**), and nitrogen retained in egg (**NRegg**) and body (**NRbody**) were calculated using equations provided by Barzegar et al. (2019) and De Cloet et al. (2023) as follows:


$$\begin{array}{l} Nitrogen\;intake\;/\;day\;=\;Daily\;hen\;feed\;intake\;in\;grams\times \left(\frac{Diet\;N}{100}\right)\\ \text{ TNR }=\text{ Nitrogen intake}-\text{nitrogen excretion}\\ \text{ NRegg }=\;1.936\;\times \text{ egg mass}\\ \text{ NRbody }=\text{ total nitrogen retained }-\text{ nitrogen retained in egg} \end{array}$$

### Economic analysis

Economic performance was evaluated at weeks 34 (Phase 1) and 44 (Phase 2) using equations stipulated by Poudel et al. (2023) and Oketch et al. (2025b). Egg income was based on mean egg weight and market price as of 1 July 2025. The prices for Jumbo (above 68 g), extra-large (60–68 g), large (60–52 g), medium (52–44 g), and small (less than 44 g) sizes were estimated to average $2.09, $1.77, $1.60, $1.30, and $1.09 per dozen eggs. Per-egg value was adjusted to hen-day egg production over 2 wk. Ingredient prices and intake per hen were used to estimate the feed costs. The difference between egg income and feed costs was considered to be the return on investment (**ROI**).

### Statistical analyses

Cages were the experimental units for laying performance, egg quality, economic performance, and nitrogen balance, while selected birds were used for tibia and abdominal fat analyses. Results are presented as means ± SEM. The data were analyzed using the General Linear Model (**GLM**) procedure for one-way ANOVA utilizing SPSS Version 29 (IBM SPSS Inc., Armonk, NY, USA). Results are presented as means, and polynomial contrasts were used to determine linear and quadratic treatment responses. Statistical significance was set at *P* < 0.05, and trends were noted at 0.05 < *P* < 0.10. When significant differences were found, means were separated using Tukey’s Multiple Range Test.

## Results

The analyzed chemical composition of the experimental diets is presented in Table 1. The analyzed CP values corresponded to the formulated target of 0.50 percentage points reduction for the four dietary protein levels that were evaluated.

### Body weight and abdominal fat contents

Hen body weight was unaffected by balanced CP reduction (*P* > 0.10), as shown in Table 2. However, linear trends for reduced body weight gain (141.66 to 95.66 g; *P* = 0.089) but improved abdominal fat contents (2.66 to 2.85%; *P* = 0.059) were observed with balanced CP reduction, particularly with the VLP series.

**Table 2.** Effects of balanced dietary CP/AA levels on the growth performance and abdominal fat contents of laying hens

Items	Diets^1^				SEM^2^	*P*-value	
	HP	MP	LP	VLP		Linear	Quadratic
**Body weight at week 26**	1797.38	1791.81	1787.50	1809.32	9.455	0.611	0.326
**Body weight at week 34**	1860.50	1853.78	1850.78	1867.54	7.465	0.783	0.427
**Body weight at week 36**	1902.63	1884.93	1882.35	1885.17	9.326	0.412	0.493
**Body weight at week 44**	1940.04	1910.07	1903.91	1904.97	17.074	0.115	0.321
**Body weight gain^3^**	141.66	118.27	116.42	95.66	19.237	0.089	0.921
**Abdominal fat contents, %**	2.66	2.61	2.73	2.85	0.228	0.059	0.288

1 HP (high protein; 18.00% and 17.00%), MP (medium protein; 17.50% and 16.50%), LP (low protein; 17.00% and 16.00%), and VLP (very low protein; 16.50% and 15.50%) for Phase 1 (week 26–34) and Phase 2 (week 36–44), respectively.

2 Pooled standard error of the mean.

3 Calculated as the difference between the final and initial hen body weight at week 44 and 26, respectively.

### Productive performance

As shown in Table 3, egg production rate, feed consumed, and egg loss percentages were unaffected (*P* > 0.10) by balanced protein reduction. However, balanced CP reduction was a limiting factor for egg weight, egg mass, and feed conversion ratio. Hens fed VLP series (15.50% and 16.50% CP) linearly reduced (*P* < 0.05) egg weights by approximately 1 to 2% across all measured timepoints (26–30, 31–34, 36–40, 41–44, and 26–44 WOA) when compared to HP. Relative to HP, egg weights with the VLP diet reduced from 57.94 to 56.65 g, 59.50 to 58.69 g, 61.61 to 60.60 g, 62.39 to 61.66 g, and 60.36 to 59.40 g across the respective periods. Indicating lowered feed conversion efficiency, the LP and VLP series led to higher FCR by approximately 2-3% (*P* < 0.05) at 31–34, 36–40, and 26–44 WOA when compared to the higher protein HP and MP series. Trends toward reduced feed conversion efficiency (*P* = 0.062) were also observed with the LP and VLP series at 26–30 WOA, with FCR values rising by approximately 2.50% from 1.97 to 2.02 g feed/g egg. Additionally, linear tendencies toward reduced egg mass were observed, particularly with the LP and VLP series at 26–30 WOA (55.43 to 52.59 g/hen/day; *P* = 0.070) and 26–44 WOA (57.02 to 55.11 g/hen/day; *P* = 0.080).

**Table 3.** Effects of balanced dietary CP/AA levels on the productive performance of laying hens

Items	Diets^1^				SEM^2^	*P*-value	
	HP	MP	LP	VLP		Linear	Quadratic
**Week 26–30**							
**Hen-day egg production %**	95.68	94.94	93.25	92.75	5.166	0.254	0.952
**Feed intake, g/d/hen**	114.35	114.80	114.73	114.62	0.752	0.874	0.743
**Feed conversion ratio, g feed/g egg**	1.97	2.00	2.02	2.02	0.084	0.062	0.637
**Egg weight, g**	57.94^b^	57.42^a,b^	56.94^a,b^	56.65^a^	2.116	0.001	0.681
**Egg mass, g/d/hen**	55.43	54.49	53.10	52.59	4.852	0.070	0.860
**Egg loss percentage, %**	2.65	2.34	2.59	2.34	0.056	0.952	1.000
**Week 31–34**							
**Hen-day egg production %**	95.59	94.89	94.10	93.70	3.140	0.580	0.955
**Feed intake, g/d/hen**	116.12	116.32	116.56	117.10	1.578	0.338	0.818
**Feed conversion ratio, g feed/g egg**	1.95^a^	1.95^a^	1.99^b^	2.00^b^	0.071	0.034	0.603
**Egg weight, g**	59.50^b^	59.45^a,b^	59.02^a,b^	58.69^a^	1.444	0.004	0.515
**Egg mass, g/d/hen**	56.83	56.41	55.52	54.98	3.150	0.336	0.966
**Egg loss percentage, %**	2.63	2.35	2.53	2.43	0.085	0.976	0.739
**Week 36–40**							
**Hen-day egg production %**	94.15	94.20	92.65	93.00	2.942	0.676	0.956
**Feed intake, g/d/hen**	117.78	117.78	118.66	117.99	1.571	0.584	0.578
**Feed conversion ratio, g feed/g egg**	1.91^a^	1.92^a^	1.95^b^	1.95^b^	0.077	0.010	0.630
**Egg weight, g**	61.61^b^	61.52^b^	60.76^a^	60.60^a^	1.921	<0.001	0.839
**Egg mass, g/d/hen**	57.99	57.91	56.30	56.36	3.511	0.364	0.964
**Egg loss percentage, %**	2.68	2.42	2.60	2.38	0.073	0.904	1.000
**Week 41–44**							
**Hen-day egg production %**	92.71	93.06	93.75	91.62	3.330	0.771	0.532
**Feed intake, g/d/hen**	118.21	118.24	118.74	118.45	0.912	0.757	0.858
**Feed conversion ratio, g feed/g egg**	1.89	1.91	1.92	1.92	0.045	0.164	0.549
**Egg weight, g**	62.39^b^	61.80^a^	61.80^a^	61.66^a^	1.219	<0.001	0.075
**Egg mass, g/d/hen**	57.83	57.51	57.94	56.50	2.440	0.518	0.647
**Egg loss percentage, %**	2.64	2.37	2.57	2.46	0.047	0.905	0.789
**Week 26–44**							
**Hen-day egg production %**	94.53	94.27	93.44	92.77	3.005	0.332	0.884
**Feed intake, g/d/hen**	116.62	116.79	117.17	117.03	0.933	0.403	0.717
**Feed conversion ratio, g feed/g egg**	1.93^a^	1.95^a,b^	1.97^b^	1.97^b^	0.071	<0.001	0.641
**Egg weight, g**	60.36^b^	60.05^b^	59.63^a^	59.40^a^	1.603	<0.001	0.683
**Egg mass, g/d/hen**	57.02	56.58	55.72	55.11	3.212	0.080	0.920
**Egg loss percentage, %**	2.65	2.37	2.57	2.40	0.065	0.906	0.792

1 HP (high protein; 18.00% and 17.00%), MP (medium protein; 17.50% and 16.50%), LP (low protein; 17.00% and 16.00%), and VLP (very low protein; 16.50% and 15.50%) for Phase 1 (week 26–34) and Phase 2 (week 36–44), respectively.

2 Pooled standard error of the mean.

a,b Means with different superscripts within the same column differ significantly.

### Internal egg and eggshell quality

Several egg quality parameters, including eggshell color, specific gravity, shell thickness, and the relative percentages of the shell, albumen, and yolk, were unaffected (*P* > 0.10) by graded levels of balanced CP reduction (Table 4). However, the lower-protein VLP series reduced Haugh units (*P* < 0.05) at week 40 by approximately 3% (92.79 to 88.85) and across the overall period from 26 to 44 WOA by approximately 1.65% (94.60 to 93.04), relative to the HP, MP, and LP series. A linear trend for reduced Haugh units (93.01 to 91.33; *P* = 0.092) was also observed with reducing dietary protein at week 34, particularly with the VLP series. Additionally, reduced dietary protein in the LP and VLP series tended to improve yolk color intensity at 44 WOA (7.94 to 8.19; *P* = 0.095) and for the overall period from 26 to 44 WOA (8.36 to 8.49; *P* = 0.076, respectively). Moreover, tendencies toward improved egg-breaking strength (5.39 to 5.51 kg; *P* = 0.058) were observed with balanced CP reduction in the LP and VLP series from weeks 26 to 44.

**Table 4.** Effects of balanced dietary CP/AA levels on egg quality of laying hens

Items	Diets^1^				SEM^2^	*P*-value	
	HP	MP	LP	VLP		Linear	Quadratic
**Week 30**							
**Egg-breaking strength, kg**	4.95	4.99	4.97	4.98	0.161	0.830	0.857
**Shell color**	29.20	28.49	28.73	29.00	2.944	0.847	0.237
**Egg specific gravity**	1.07	1.09	1.06	1.06	0.158	0.209	0.596
**Shell thickness, µm**	358	362	361	359	0.025	0.992	0.453
**Albumen height, mm**	9.21	9.09	9.16	9.19	0.522	0.977	0.596
**Haugh Units**	96.69	95.47	95.63	95.84	5.172	0.498	0.366
**Yolk color**	8.64	8.60	8.62	8.73	0.554	0.231	0.150
**Shell percentage, %**	13.36	13.34	13.44	13.41	0.452	0.666	0.982
**Yolk percentage, %**	27.32	27.48	27.15	27.30	1.338	0.913	0.860
**Albumen percentage, %**	59.38	58.38	58.68	58.80	1.997	0.390	0.885
**Week 34**							
**Egg-breaking strength, kg**	5.51	5.52	5.61	5.60	0.509	0.187	0.845
**Shell color**	27.98	28.68	28.22	28.48	2.889	0.618	0.635
**Egg specific gravity**	1.09	1.07	1.08	1.09	0.086	0.989	0.716
**Shell thickness, µm**	351	354	357	353	0.019	0.610	0.287
**Albumen height, mm**	8.51	8.44	8.50	8.28	0.963	0.407	0.691
**Haugh Units**	93.01	93.79	92.26	91.33	9.923	0.092	0.323
**Yolk color**	8.42	8.46	8.37	8.49	0.494	0.666	0.439
**Shell percentage, %**	13.54	13.31	13.36	13.32	1.023	0.385	0.557
**Yolk percentage, %**	27.06	26.89	26.66	26.73	1.669	0.473	0.745
**Albumen percentage, %**	59.77	59.31	59.21	59.42	2.311	0.590	0.488
**Week 40**							
**Egg-breaking strength, kg**	5.54	5.60	5.77	5.66	0.941	0.139	0.304
**Shell color**	28.97	28.56	28.77	28.87	1.703	0.958	0.613
**Egg specific gravity**	1.09	1.07	1.07	1.09	0.089	0.982	0.741
**Shell thickness, µm**	354	357	350	354	0.056	0.782	0.785
**Albumen height, mm**	8.94	9.11	9.02	8.69	0.676	0.208	0.107
**Haugh Units**	92.01^b^	92.79^b^	92.26^b^	88.85^a^	4.109	0.015	0.023
**Yolk color**	8.42	8.44	8.50	8.54	0.523	0.384	0.918
**Shell percentage, %**	13.52	13.48	13.32	13.31	1.026	0.216	0.925
**Yolk percentage, %**	27.51	27.88	28.16	27.32	1.842	0.151	0.730
**Albumen percentage, %**	59.19	59.11	59.27	59.42	1.245	0.755	0.850
**Week 44**							
**Egg-breaking strength, kg**	5.56	5.56	5.70	5.60	0.608	0.438	0.476
**Shell color**	28.97	28.56	28.77	28.87	1.699	0.977	0.650
**Egg specific gravity**	1.08	1.06	1.07	1.08	0.110	0.949	0.695
**Shell thickness, µm**	364	357	366	371	0.026	0.957	0.772
**Albumen height, mm**	9.66	9.42	9.43	9.49	0.975	0.487	0.373
**Haugh Units**	96.69	95.14	94.65	96.10	6.384	0.490	0.145
**Yolk color**	8.01	7.94	8.14	8.19	1.080	0.095	0.474
**Shell percentage, %**	13.65	13.57	13.62	13.40	1.043	0.362	0.664
**Yolk percentage, %**	27.92	28.21	28.43	28.56	1.651	0.130	0.809
**Albumen percentage, %**	58.76	58.91	58.64	59.05	1.693	0.818	0.829
**Week 26–44**							
**Egg-breaking strength, kg**	5.39	5.41	5.51	5.48	0.504	0.058	0.270
**Shell color**	28.73	28.57	28.64	28.80	1.076	0.932	0.560
**Egg specific gravity**	1.08	1.07	1.07	1.08	0.063	0.911	0.760
**Shell thickness, µm**	357	359	358	363	0.029	0.945	0.498
**Albumen height, mm**	9.08	9.02	9.03	8.91	0.640	0.143	0.777
**Haugh Units**	94.60^b^	94.30^b^	93.66^a,b^	93.04^a^	6.616	0.003	0.693
**Yolk color**	8.38	8.36	8.41	8.49	0.552	0.076	0.268
**Shell percentage, %**	13.51	13.43	13.44	13.36	0.611	0.351	0.945
**Yolk percentage, %**	26.93	27.11	27.10	27.23	1.173	0.361	0.896
**Albumen percentage, %**	59.03	58.93	58.95	59.17	1.043	0.786	0.676

1 HP (high protein; 18.00% and 17.00%), MP (medium protein; 17.50% and 16.50%), LP (low protein; 17.00% and 16.00%), and VLP (very low protein; 16.50% and 15.50%) for Phase 1 (week 26–34) and Phase 2 (week 36–44), respectively.

2 Pooled standard error of the mean.

a,b Means with different superscripts within the same column differ significantly.

### Mineral utilization

Tibia weight, length, volume, and density were unaffected (*P* > 0.10) by dietary treatments (Table 5). However, tibia-breaking strength tended to decline (*P* = 0.094) with graded dietary protein reduction, decreasing from 27.62 kg in HP to 25.54–25.68 kg in the LP and VLP diets.

**Table 5.** Effects of balanced dietary CP/AA levels on some tibia characteristics of laying hens

Items	Diets^1^				SEM^2^	*P*-value	
	HP	MP	LP	VLP		Linear	Quadratic
**Tibia weight, g**	15.10	14.04	14.47	14.65	0.072	0.972	0.367
**Tibia length, cm**	12.78	12.37	12.33	12.42	0.085	0.863	0.867
**Tibia volume, cm³**	9.44	9.61	9.39	9.48	0.166	0.681	0.790
**Tibia density, g/cm³**	1.60	1.46	1.54	1.55	0.045	0.734	0.384
**Tibia breaking strength**	27.62	26.70	25.54	25.68	2.822	0.094	0.877

1 HP (high protein; 18.00% and 17.00%), MP (medium protein; 17.50% and 16.50%), LP (low protein; 17.00% and 16.00%), and VLP (very low protein; 16.50% and 15.50%) for Phase 1 (week 26–34) and Phase 2 (week 36–44), respectively.

2 Pooled standard error of the mean.

### Nitrogen balance

Total nitrogen retained and nitrogen retained in either egg or body were unaffected (*P* > 0.10) by dietary protein levels (Table 6). Relative to the HP diet, the VLP diet reduced nitrogen intake by approximately 13.2% (3.48 to 3.02) and 11.9% (3.45 to 3.04) at weeks 30 and 40, respectively (*P* < 0.05). Correspondingly, nitrogen excretion also declined by 17.1% (1.87 to 1.55) at week 30 and 11.8% (1.69 to 1.49) at week 40 (*P* < 0.05). Reducing dietary protein tended to lower nitrogen retained in egg (1.12 to 1.05; *P* = 0.072), particularly with the VLP series at week 40.

**Table 6.** Effects of balanced dietary CP/AA levels on nitrogen balance of laying hens

	Diets^1^				SEM^2^	*P*-value	
Items	HP	MP	LP	VLP		Linear	Quadratic
**Week 30 (grams/hen/day)**							
**N intake^3^**	3.48^c^	3.30^b^	3.14^a,b^	3.02^a^	0.607	<0.001	0.456
**N excreted**	1.87^b^	1.85^b^	1.73^ab^	1.55^a^	0.438	<0.001	0.153
**Total N retained^4^**	1.61	1.45	1.41	1.47	0.261	0.105	0.398
**N retained in egg^5^**	1.06	1.02	1.01	1.01	0.063	0.277	0.446
**N retained in body^6^**	0.55	0.43	0.40	0.45	0.200	0.284	0.213
**Week 40 (grams/hen/day)**							
**N intake**	3.45^c^	3.36^b^	3.19^ab^	3.04^a^	0.544	<0.001	0.547
**N excreted**	1.69^b^	1.61^b^	1.56^ab^	1.49^a^	0.308	0.012	0.758
**Total N retained**	1.76	1.75	1.63	1.55	0.255	0.105	0.911
**N retained in egg**	1.12	1.10	1.09	1.05	0.045	0.072	0.502
**N retained in body**	0.64	0.65	0.54	0.50	0.217	0.184	0.784

1 HP (high protein; 18.00% and 17.00%), MP (medium protein; 17.50% and 16.50%), LP (low protein; 17.00% and 16.00%), and VLP (very low protein; 16.50% and 15.50%) for Phase 1 (week 26–34) and Phase 2 (week 36–44), respectively.

2 Pooled standard error of the mean

3 Intake was calculated as follows: Daily hen feed intake in grams × (Diet N/100).

4 Difference between N intake and excreted.

5 Calculated as egg mass (g/hen/d) × N concentration in eggs assumed as 1.936% (Barzegar et al., 2019); 1.936 is derived from the determined protein content of an egg at around 12.10 (Miranda et al., 2015).

6 Nitrogen retained in the body (g/hen/d) calculated as the difference between total N retained and N retained in the egg.

a, b, c Means with different superscripts within the same column differ significantly.

### Economic performance

As shown in Table 7, increasing protein in the HP and MP diets led to linearly improved egg income (*P* < 0.05) at 34 and 44 WOA when compared to LP and VLP diets. At 34 WOA, relative egg income decreased from $2.00 to 1.98 for the HP and MP diets to $1.78 to 1.77 for the LP and VLP diets. At 44 WOA, egg income estimates reduced from $1.96 for both HP and MP diets to $1.94–1.89 for the lower-protein LP and VLP diets. Conversely, trends for reduced feed costs ($0.54 to 0.52; *P* = 0.088) were noticed with dietary protein reduction, particularly in the LP and VLP series at week 34. However, the estimates for potential return on investment were unaffected by dietary protein levels at both 34 and 44 WOA (*P* > 0.10).

**Table 7.** Effects of balanced dietary CP/AA levels on the relative economic performance of laying hens

Items	Diets^1^				SEM^2^	*P*-value	
	HP	MP	LP	VLP		Linear	Quadratic
**Week 34 (USD)**							
**Egg income^3^**	2.00^b^	1.98^a,b^	1.78^a^	1.77^a^	0.032	0.013	0.104
**Feed cost^4^**	0.54	0.53	0.52	0.52	0.457	0.088	0.112
**Return on investment^5^**	1.47	1.45	1.25	1.25	0.437	0.107	0.112
**Week 44 (USD)**							
**Egg income**	1.96^b^	1.96^a,b^	1.94^a^	1.89^a^	0.032	0.009	0.405
**Feed cost**	0.55	0.54	0.54	0.54	0.114	0.656	0.056
**Return on investment**	1.41	1.42	1.40	1.38	0.110	0.821	0.157

1 HP (high protein; 18.00% and 17.00%), MP (medium protein; 17.50% and 16.50%), LP (low protein; 17.00% and 16.00%), and VLP (very low protein; 16.50% and 15.50%) for Phase 1 (week 26–34) and Phase 2 (week 36–44), respectively.

2 Pooled standard error of the mean.

3 Egg income was calculated using hen-day egg production, average egg weight, and the price of an egg over 2 wk.

4 Feed costs were estimated based on local ingredient prices and the average feed intake per hen over 2 wk.

5 Calculated as the difference between egg income and feed cost. a,bMeans with different superscripts within the same column differ significantly.

## Discussion

Amid growing pressure to use limited feed resources efficiently, the poultry industry continually explores strategies to enhance animal performance, health, and welfare while reducing feed costs and environmental impact from nutrient excretion. Among these strategies, reduced protein diets that have been supplemented with indispensable AA to meet hen requirements have emerged as a promising approach. Dietary CP and AA levels are regularly revised by breeding companies; however, limited data exist on whether the currently proposed specifications (Hy-Line Brown Commercial Layers, 2024) can be reduced in hen diets. The current study, therefore, investigated the capacity of graded dietary protein reduction at balanced AA levels to maintain hen performance and egg quality, modulate tibia characteristics, reduce feed costs, and lower environmental stress due to nutrient excretion from 26 to 44 WOA. Results showed that several growth and productive performance parameters, including hen body weight, egg production rate, feed intake, and egg loss percentages, were unaffected by dietary treatments. Presently, hens adjusted their feed intake accordingly to meet energy demands, maintaining growth and egg production, in line with earlier findings that low-protein diets supplemented with crystalline AA can maintain performance (Cabezas-Garcia et al., 2022; Oluwabiyi et al., 2022; Heo et al., 2023).

However, reducing dietary protein limited egg weight, egg mass, and feed conversion efficiency, consistent with previous studies (Dong et al., 2017; Barbosa et al., 2020; Oketch et al., 2025b). These negative effects are likely to be due to decreased protein-to-energy ratios, resulting in an imbalance of lower protein per calorie consumed (Woyengo et al., 2023). Thus, although hens fed low-CP diets maintained egg production rate, this effect was seemingly achieved at the expense of reduced egg weight, consequently leading to lower feed conversion efficiency. Suggesting further evidence of compromised energy-to-protein balance, we observed tendencies for improved abdominal fat contents but relatively lowered body weight gain with further reductions in dietary CP, particularly, the very low protein series. This increase in fat deposition is likely a direct consequence of excess energy spared from lowered deamination and transamination processes with further protein reduction (Cowieson et al., 2020; Heo et al., 2023; Woyengo et al., 2023). While higher fat deposition may reflect energy status more than egg production over a short-term period (Lyons et al., 2023), excessive fat accumulation from extended laying could compromise egg production and quality (Fouad and El-Senousey, 2014). In line with contemporary breeding targets, the long-term capacity of reduced CP diets to sustain laying performance (up to 100 WOA with 450–500 eggs per laying cycle) warrants further studies beyond the current trial period.

As to egg quality, balanced protein reduction did not affect shell color, specific gravity, shell thickness, albumen height, or the relative percentages of shell, yolk, and albumen. However, reducing dietary protein was a limiting factor for Haugh units, consistent with previous findings (Heo et al., 2023; Poudel et al., 2023; Oketch et al., 2025b). Variations in albumen height for egg weight are adjusted in Haugh unit calculations. The observed decline in Haugh units with low-CP diets aligns with reduced egg weights, highlighting the link between protein intake and internal egg quality. Dietary protein reduction also tended to improve yolk color, likely due to increased levels of xanthophyll-containing corn (Heo et al., 2023). Additionally, unlike previous studies that reported no effect of dietary protein reduction on shell breaking strength (Heo et al., 2023; Ali et al., 2025), our current results showed trends for improved shell breaking strength at reduced protein levels. One possible explanation for our observation is that lowering dietary protein regulates egg weight by preventing excessive egg size increase (Robinson and Kiarie, 2019). This moderation in egg weight may help preserve shell quality, as reflected in the higher percent shell relative to egg weight often observed in smaller eggs (Kim et al., 2005; Oketch et al., 2025c). Interestingly, we previously determined that egg weight exhibited a very low negative correlation to shell-breaking strength (Oketch et al., 2025c). This indicates that while reduced egg size may partly explain the improvement, additional mechanisms beyond egg size regulation are likely involved in enhancing shell strength under low-protein diets. Nevertheless, the impact of dietary protein levels on eggshell quality remains limited and inconsistent. Zhang et al. (2025) reported trends for linearly improved shell breaking strength when hens were fed higher dietary protein levels from 49 to 61 WOA. Further investigation is necessary to clarify how dietary protein level modulates shell quality and to elucidate the underlying mechanisms that could be involved.

As breeding targets are increasingly focused on extended laying, maintaining hen bone health is essential for ensuring hen welfare, performance, and egg quality (Olgun and Aygun, 2016; Alfonso-Carrillo et al., 2021). Hens possess three primary bone types: cortical, trabecular, and medullary. Medullary bone, which develops at sexual maturity in laying hens, serves as a readily available calcium reservoir during intense eggshell calcification, cushioning against dietary calcium deficiency (Toscano et al., 2020). The tibia, the second largest reserve of the medullary bone (Hanlon et al., 2022), was targeted to evaluate skeletal responses to varied levels of balanced dietary protein. The results revealed that tibia morphological traits, including weight, length, volume, and density, were unaffected by the dietary treatments. However, tendencies for reduced tibia-breaking strength were observed with balanced dietary protein reduction, suggesting a modest compromise on bone mechanical integrity and function. Previous studies on the specific influence of dietary protein level on hen bone parameters have been inconsistent. The indirect effect of reduced egg size from lowering dietary protein has been suggested to improve the calcium and phosphorus utilization, bone health, and mineral balance of laying hens (Rennie et al., 1997). For instance, Hassan et al. (2013) reported that hens fed 16% CP at 2775 kcal/kg had slightly stronger bones at 46 WOA when compared to those fed 17% CP at higher (2800 kcal/kg) or lower (2750 kcal/kg) energy levels. In contrast, studies by Dao et al. (2023), Heo et al. (2023), and Oluwabiyi et al. (2022) reported that tibia traits were unaffected by dietary protein levels.

A key finding of this study is the potential influence of dietary protein levels on mineral metabolism, as reflected in the divergent eggshell and tibia bone responses. Reduced dietary protein tended to improve egg-breaking strength but also led to a linear decline in bone strength. Similar compromise in tibia bone integrity and breaking strength has been previously reported in broilers fed low-protein diets (Cowieson et al., 2020). As to laying hens, evidence suggests that egg production is the primary driver of structural bone loss (Paneru et al., 2026), and improved shell quality, as observed in the current study, is likely to be achieved at the expense of bone health, as alluded to in previous literature (Kim et al., 2012; Alfonso-Carrillo et al., 2021). Such short-term tradeoff in mineral utilization may increase susceptibility to fractures during sustained laying. This pattern could mirror observations in human osteoporosis, where low protein intake is associated with increased bone loss (Kerstetter et al., 2011; Mangano et al., 2014). Nevertheless, research on the effects of dietary protein levels on bone function in laying hens remains limited, and the findings available so far are inconclusive. Given the dynamic remodeling of the tibial medullary bone under the hormonal influence of estrogen, future studies should elucidate the impact of dietary protein reduction on bone parameters and reconcile those effects to shell quality.

Regarding the impact of balanced dietary protein reduction on the economic and environmental sustainability of hen production, we assessed the nitrogen balance and cost-benefit analyses. In line with previous studies (Lee et al., 2024; Liu et al., 2024; Oketch et al., 2025b), it was observed that balanced dietary protein reduction led to lowered nitrogen excretion by approximately 10%, indicating improved nitrogen utilization and reduced environmental nitrogen load. Reducing dietary protein also led to trends toward reduced relative nitrogen content retained in egg, suggesting reduced protein synthesis efficiency and deposition with reduced protein diets (Li et al., 2024). Regarding economic performance, while increasing dietary protein led to improved egg income, further reductions in dietary protein resulted in relatively lower feed costs. Of particular interest, increments in potential income from higher egg weights did not offset the increased feed costs from higher dietary protein provision; thus, the potential return on investment was unaffected by dietary protein levels. This observation suggests that feeding higher-protein diets to maximize egg weight and performance may offer a limited economic advantage, especially when considering overall profitability (Shim et al., 2013).

Under the conditions of this study, we conclude that balanced dietary protein reduction by up to 1.50 percentage points from weeks 26 to 44 maintained hen productive performance and most egg quality traits while substantially reducing nitrogen excreted and relative feed costs. These findings provide important insights for lowering feed costs and maintaining hen performance while improving the environmental and economic sustainability of poultry farming. Given the current breeding target of extended laying cycles, the long-term effects of balanced protein reduction beyond the current test period warrant further investigation. Divergent effects of dietary protein levels on mineral utilization were also observed on tibia traits and eggshell quality, highlighting the need for further research on how dietary protein influences laying hen mineral digestibility, deposition, and utilization; reproductive hormonal profiles; eggshell quality; and bone health and function beyond the current experimental period. Further research on the influence of long-term abdominal fat deposition with reduced dietary protein on hen body composition, energy status, laying performance, and egg quality over longer laying cycles is also suggested.

## Abbreviations:

AA: amino acids
CP: crude protein
FCR: feed conversion ratio
GLM: general linear model
HDEP: hen-day egg production
HP: high protein
LP: low protein
MP: medium protein
NRbody: nitrogen retained in body
NRegg: nitrogen retained in egg
PRAL: potential renal acid load
ROI: return on investment
TNR: total nitrogen retained
VLP: very low protein
WOA: weeks of age
